# Immersive Virtual Reality for Pain and Relaxation in Older Adults Following Elective Inpatient Abdominal Surgery: Single-Arm Study Examining Feasibility and Acceptability

**DOI:** 10.2196/81791

**Published:** 2026-04-17

**Authors:** Christina Keny, Ujala Shafiq, Karl Lorenz, Marcia Russell, Heather Leutwyler, Laura M Wagner, Victoria Tang, Linda G Park

**Affiliations:** 1 Department of Community Health Systems, School of Nursing, University of California, San Francisco, Box 0608, 490 Illinois Street, #93P, San Francisco, CA, 94158, United States, 1 4086126143; 2 Department of Primary Care and Population Health (PCPH), Stanford University School of Medicine, Palo Alto, CA, United States; 3 Department of Surgery, David Geffen School of Medicine at UCLA, Los Angeles, CA, United States; 4 Department of Medicine, Division of Geriatrics, University of California, San Francisco, San Francisco, CA, United States

**Keywords:** geriatrics, surgery, pain, virtual reality, postoperative pain, geriatric surgery

## Abstract

**Background:**

There is mounting evidence to suggest that immersive virtual reality (IVR) can improve pain in older adults in community settings, yet the use of IVR postoperatively in the acute postoperative period following major elective abdominal surgery remains largely underexplored.

**Objective:**

This single-arm pilot study aimed to assess the feasibility, acceptability, and preliminary impact of IVR on self-reported postoperative pain and relaxation levels in older adults following elective major abdominal surgery.

**Methods:**

We recruited individuals aged 55 years and older undergoing elective abdominal surgery at an academic medical center from October 2023 to February 2024. We evaluated feasibility through accrual rate, intervention completion, and questionnaire compliance; acceptability via the System Usability Scale (SUS) and a user experience survey; and tolerability by monitoring self-reported side effects. The preliminary impact of IVR on self-reported pain intensity and relaxation levels was assessed through pre- and postintervention comparisons.

**Results:**

A total of 29 participants, with a median age of 73 (IQR 55‐81) years, were enrolled and completed at least 1 IVR session, with 19 also completing a second session. Perceived usability and overall acceptance of IVR were high, with minimal side effects reported. In terms of the preliminary impact of IVR, statistically significant improvements were observed in both pain and relaxation levels from pre- to post-IVR on day 1 and day 2.

**Conclusions:**

This study suggests the feasibility and acceptability of IVR as a potential future intervention for postoperative pain management and enhancing relaxation among older adults following elective inpatient abdominal surgery. The preliminary findings suggest the need for large-scale studies across additional complex inpatient abdominal surgeries to confirm the acceptance and efficacy of IVR as a postoperative pain management intervention across a wide range of diverse older demographics. Future research is critical to evaluating the therapeutic potential of IVR in a variety of surgical and patient-specific contexts.

## Introduction

Nearly 4 million operations are performed annually on individuals aged 65 years or older in the United States, a number that is expected to rise significantly as the population continues to age [1,2]. Optimal pain management following major surgery is crucial for older adults who face unique risks associated with uncontrolled postoperative pain, such as delirium, functional decline, and reduced psychosocial well-being [3-6]. Effectively managing pain in older surgical adults is often complicated by challenges stemming from age-related physiological changes, the presence of multiple comorbidities, and the intricacies of polypharmacy [7,8]. Moreover, older adults have an increased risk of developing opioid-related adverse events, addiction, and/or chronic pain following surgery when compared to younger age groups [9-12]. These factors may contribute to the heightened risks associated with traditional pharmacologic pain management in the older adult [13-15].

Given the complexities and inherent risks in managing acute postoperative pain in this demographic, there is a growing interest in exploring innovative nonpharmacological methods for pain [16-18]. Among these emerging solutions, immersive virtual reality (IVR) has garnered significant interest from both the clinical and research communities [19]. Defined by the use of a head-mounted display (HMD) with motion tracking capabilities, IVR effectively provides users with a believable sense of reality while engaged in a virtual environment [20]. This profound sense of “presence” within the virtual environment often provides positive distraction away from pain stimuli [21-23].

Research on IVR has shown significant potential in reducing acute pain across various clinical settings, such as burn wound care [24,25]. Recent studies have also demonstrated that IVR is effective in reducing postoperative pain in pediatric and young to middle-aged adults following various surgical procedures [25,26]. Contrary to the common belief that older adults are hesitant to embrace new technologies, there is mounting evidence to suggest that using IVR for pain management in the older adult demographic is promising [27-30]. Research focused on IVR use for chronic pain management in community-dwelling older adults has demonstrated improved pain tolerance with a high degree of acceptance as part of pain management [31,32]. Finally, studies have also indicated the acceptability and efficacy of IVR use for postoperative pain among older adults, specifically in elective total knee arthroplasty operations [33,34].

It is key to recognize that the user experience of IVR for postoperative pain management may differ across various older adult subgroups and types of surgical procedures [30,35]. Appreciating these distinctions is vital for tailoring IVR applications to effectively manage pain in a wide range of surgical scenarios and across different older age demographics. Thus, IVR could serve as a promising alternative or adjunct to traditional pain management techniques. However, the feasibility, acceptability, and tolerability of IVR for postoperative pain among older adults across a spectrum of major surgical procedures, including complex abdominal operations, remains largely underexplored. Therefore, the aim of this single-arm study was to investigate the initial feasibility and acceptability (primary outcome), and the preliminary impact (secondary outcome) of IVR on postoperative pain and relaxation levels in older adults during the initial days following inpatient elective abdominal surgery.

## Methods

### Study Design

This study used a prospective pretest-posttest single-arm study design to ascertain the feasibility, acceptability, and preliminary impact of IVR on pain and relaxation outcomes among older adults following elective major abdominal surgery. The participant enrollment sample size was set for practical reasons and not driven by power analysis for this initial study [36]. This study followed the CONSORT (Consolidated Standards of Reporting Trials) extension to pilot and feasibility studies statement, which is recommended for adaptation in nonrandomized feasibility studies [37]. This study design also aligns with the recommendations and methodological framework proposed by the Virtual Reality Clinical Outcomes Research Experts (VR-CORE) on best practices for the development and testing of IVR treatments in clinical care [38].

### Ethical Considerations

The study received approval from the University of California, San Francisco Institutional Review Board (IRB #19‐28391) and is registered under clinicaltrials.gov (NCT06095661). All participants were provided with written and verbal informed consent before taking part in the study. Data were stored on secure, password‑protected servers, and all analyses used deidentified data in accordance with the approved protocol. All responses to study questionnaires were deidentified and entered directly into an electronic tablet that was password-protected. A one-time US $25 gift card was provided to all participants for their participation in at least 1 IVR session. A subgroup of participants was additionally offered the option to complete a single user experience survey prior to hospital discharge. Those who opted to participate in the survey received an additional US $25 gift card.

### Participants

We used purposeful sampling to recruit adults aged ≥55 years undergoing elective inpatient abdominal surgery at the University of California, San Francisco, Colorectal and General Surgery clinics. Those who were potentially eligible for the study and who were interested in participation were screened via telephone. Inclusion criteria were individuals anticipated to have an elective abdominal operation requiring hospitalization for at least 48 hours after surgery and those who were able to speak and write in English. Exclusion criteria encompassed individuals with a reported history of self-reported motion sickness, severe cognitive impairment, epilepsy, eye, neck, or face injuries, blindness or severe visual impairment, severe hearing loss, or acute illness hindering postsurgery IVR use. Participants with immediate preintervention nausea, vomiting, or dizziness were also excluded. All participants continued to receive their usual surgical care as per the recommendations of clinical providers and were not asked to decline or change any adjunct strategies for pain management, as the intent of the study was to understand the initial feasibility and acceptability of IVR during the first days after surgery on an inpatient hospital unit.

### Intervention

This study used the REAL System i-Series IVR HMD from Penumbra, Inc, featuring built-in audio and gaze-controlled navigation [39]. This IVR system, preloaded with various 360-degree immersive environments for positive distraction and relaxation, includes experiences such as mindful meditation, travel, nature scenes, and games. The IVR system provides motion tracking through sensors embedded in the headset, capturing all possible participant movements, thus facilitating an extensive immersive experience. The decision to leverage this specific IVR device was 2-fold: (1) the HMD unit was preloaded with a built-in library of experiences, allowing the user access to a wide variety of IVR-positive distraction environments, and (2) the device offered gaze-controlled navigation, potentially allowing ease of use in the immediate postoperative phase of care following major abdominal surgery.

### Procedure

During the preoperative surgery visit, clinical staff provided possible participants with an informational flyer and an email introducing the study. Participant information was gathered from the electronic medical record, and potential participants were then contacted by phone and screened for eligibility by the research team. Screening included assessment of cognitive function using the Short Portable Mental Status Questionnaire (SPMSQ), self-reported history of motion sickness, epilepsy, blindness, severe hearing deficits, and any current eye, face, or neck injuries. If deemed eligible and interested in study participation following the initial screening, participants electronically received the informed consent form. Once the consent form was signed, participants were asked to electronically complete an online questionnaire for sociodemographic and clinical data prior to their date of surgery. Prior to surgery, participants received an instructional video link demonstrating the use of the IVR headset, along with a catalog of available immersive content options. Immediately before each intervention session, the study team again reviewed headset use and content selection with the participant. A trained study team member with expertise in the IVR protocol remained present throughout the session to assist with setup, navigation, and troubleshooting as needed.

All participants enrolled in the study were provided with the opportunity to engage in at least 1 IVR session within their hospital room. These sessions were made available starting from the next day following surgery and could extend up to the second day after surgery, ensuring a maximum offering of 2 IVR sessions in total. The IVR intervention was administered to the patient in a seated or lying position by a member of the research team, who was present during and up to 15 minutes after each IVR session. Participants then choose their desired experience within the IVR content library. IVR program preference selection and length of the session were determined by the participant, up to a maximum of 30 minutes per session.

Immediately before and after the IVR intervention, participants reported their pain intensity level and state of relaxation on an 11-point Numeric Rating Scale (NRS) ranging from “0” representing “no pain” or “not relaxed at all” to “10” representing “pain as bad as you can imagine” or “as relaxed as possible.” Adverse outcomes were assessed up through the first 15 minutes after each IVR session using an adapted 4-item Simulator Sickness Questionnaire (SSQ) [40]. All responses were deidentified and entered directly into an electronic tablet that was password-protected.

### Outcome Measures

#### Acceptability

In the context of this study, acceptability refers to participants’ willingness to use IVR in the initial 2 days of their hospitalization and their ability to tolerate IVR use, with minimal side effects reported. Acceptability was assessed by the System Usability Scale (SUS) in all participants (N=29), with a subgroup of 21 participants also completing a user experience survey. After at least 1 session of IVR, all participants were asked to complete the SUS once. SUS is considered a valid and reliable instrument for measuring perceived usability of a technology system and consists of a 10-item questionnaire with 5 response options, with “1” representing “strongly disagree” and “5” representing “strongly agree” [41]. A higher score indicates greater self-reported usability, reflecting a positive attitude toward using the system [42]. An 8-item user experience survey created by the research team using a 5-point Likert scale, ranging from 1 (“totally disagree”) to 5 (“totally agree”), was also administered to quantify participant satisfaction with IVR. The scores from the user experience survey for each sentiment level (strongly disagree-1, disagree-2, neutral-3, agree-4, and strongly agree-5) are presented as a mean and SD. Tolerability refers to the evaluation of adverse events that occurred as a result of IVR use, related to either the hardware or software components [38]. Adverse outcomes, which include symptoms such as nausea, headache, blurred vision, and dizziness, were assessed using a 4-item adapted questionnaire of the SSQ [40]. This questionnaire was administered immediately after each IVR session, allowing participants to indicate the presence or absence of these symptoms with a “Yes” or “No” response. Participants also had the option to free-text any side effects they felt occurred during or immediately after IVR use.

#### Feasibility

In this study, feasibility is defined as the extent to which potentially eligible participants consented to join the study during the recruitment phase and the degree to which enrolled participants successfully completed the IVR intervention and all questionnaires. To evaluate feasibility, we measured the rate of participant accrual, reasons for nonparticipation, the successful completion of the intervention on the first and second days after surgery, and the mean duration time spent using IVR during each session. We also captured reasons for not completing a second IVR session.

We also evaluated the feasibility by the rate at which participants completed baseline questionnaires. The baseline characteristics captured through questionnaires were self-reported perceived health status, anxiety, depression, and pain catastrophizing prior to the operation. Perceived health status was measured using the EQ-5D-5L questionnaires’ Visual Analog Scale (VAS), with scores ranging from 0 to 100, with a higher score indicating higher perceived health [43,44]. Anxiety was measured using the Generalized Anxiety Disorder-7 (GAD-7) scale, with higher scores indicating higher anxiety levels (total score for GAD is 0‐21) [45]. Depression was assessed using the Patient Health Questionnaire-8 (PHQ-8), which ranges from 0 to 24, with a higher score indicating higher levels of depression [46]. Finally, we leveraged the Pain Catastrophizing Scale (PCS), where higher scores signify more intense negative thoughts and feelings toward pain [47].

#### Preliminary Clinical Impact on Pain and Relaxation

Preliminary clinical impact of IVR on pain intensity levels and state of relaxation was measured through pre- and postintervention mean differences using independent paired sample *t* tests if the data were deemed normal. Nonparametric continuous data were evaluated using the Wilcoxon signed-rank test. Pain intensity level was measured on an 11-point NRS and ranged from “0” representing “no pain” to “10” representing extreme pain. State of relaxation is also measured on an 11-point NRS with “0” representing “not relaxed at all” to “10” representing “as relaxed as one could imagine.” Both pain and relaxation were assessed immediately prior to and after each IVR session.

### Data Analysis

Acceptability, feasibility, and tolerability are reported as descriptive statistics. User experience surveys for each sentiment level were presented as a mean and SD. Prior to analyzing the preliminary effects of the IVR intervention, we assessed the normality of the distribution of pain and relaxation levels using the Shapiro-Wilk test. Normal data were compared as pre-post mean differences with independent paired 2-sample *t* tests. Nonparametric data were reported as a median and compared using the Wilcoxon signed-rank test. The significance level was set at *P*<.05. All statistical analyses were performed using the STATA statistical software, version 18 SE (StataCorp LLC) [48].

## Results

### Characteristics of Participants

**Figure 1.**
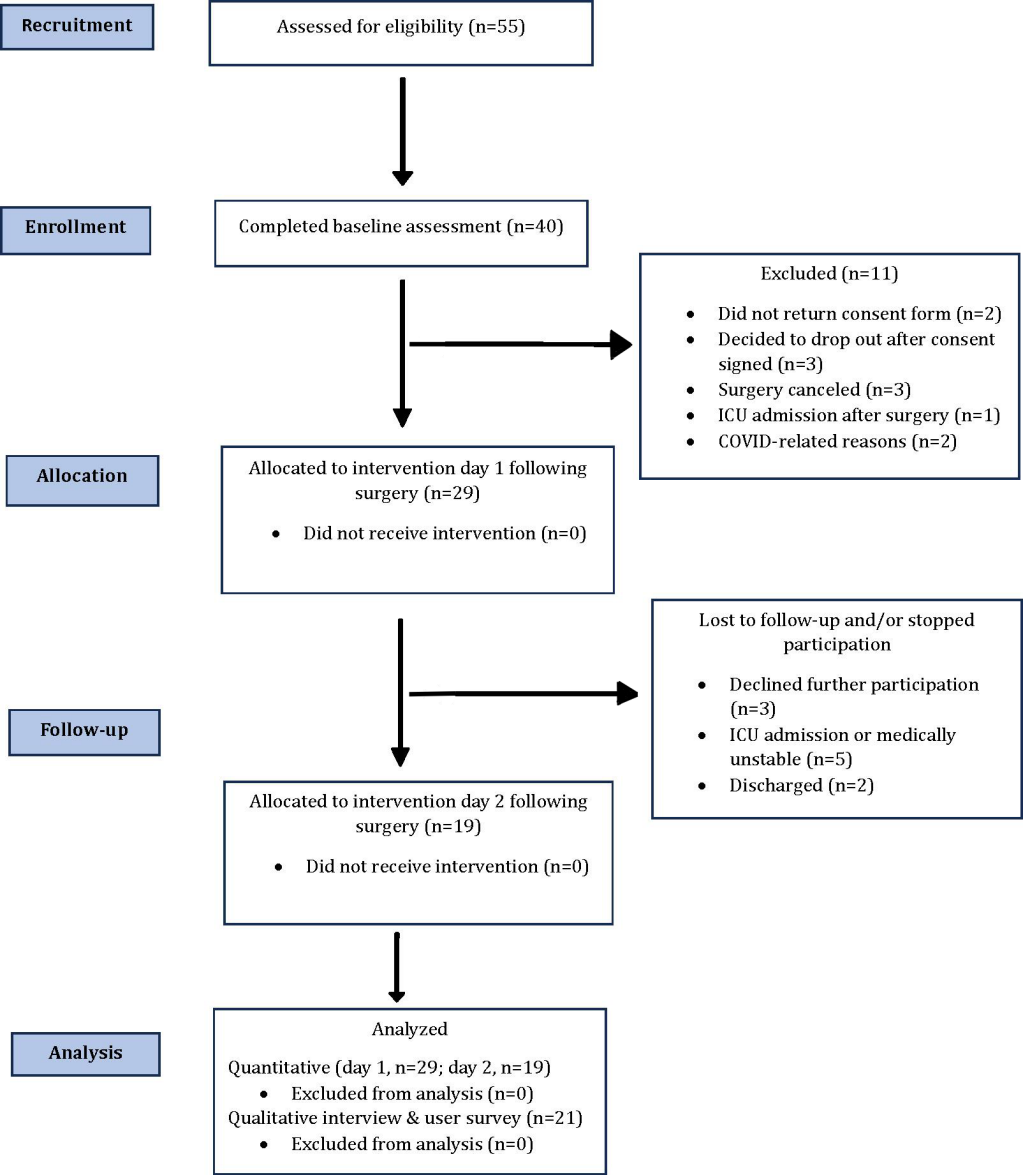
CONSORT (Consolidated Standards of Reporting Trials) flow diagram.

Fifty-five participants scheduled for elective inpatient abdominal surgery were assessed for study eligibility between October 2023 and February 2024 (Figure 1). Among possible participants, 15 in total were excluded due to not meeting the inclusion criteria. A total of 40 participants completed the baseline questionnaires prior to surgery. Of the 40 participants enrolled, 11 withdrew for several reasons, including failure to complete the written informed consent prior to surgery (n=2). Postoperative consent was not pursued in these cases because participants had undergone general anesthesia. Additional reasons for withdrawal included surgery cancellation (n=3), postoperative Intensive Care Unit admission (n=1), and testing positive for COVID-19 following surgery (n=2). Three additional participants withdrew after providing consent for unknown reasons. A total of 29 enrolled participants were allocated to and completed the first IVR intervention the next day following their surgery, with 19 (65.5%) additionally completing a second intervention the next day. Most participants reported no prior experience with IVR (27/29, 93.1%).

Among 29 participants, the median age was 73 (IQR 55-81) years. A total of 79.3% (23/29) identified as White, 62.10% (18/29) were female, and all but 2 participants reported at least some level of college education (27/29, 93.1%; Table 1). Nearly half of the participants (14/29, 48.3%) underwent a low anterior resection abdominal operation, with cancer as the most common indication for surgery (19/29, 65.5%). More than half of participants reported pain in the 2 weeks prior to surgery (16/29, 55.2%), with a few individuals taking opioid medications for pain (n=3). In our evaluation of the baseline characteristics of the study sample, we found that participants typically described their perceived health status as generally good. Additionally, they reported experiencing mild levels of anxiety and depression, alongside minimal tendencies toward pain catastrophizing, before undergoing surgery and the IVR intervention (Table 1).

**Table 1.** Sociodemographic, baseline characteristics, and clinical descriptives (N=29).

Characteristics	Values
Age (years), median (range)	73 (55‐81)
Sex, n (%)	
Male	11 (37.9)
Female	18 (62.1)
Race or ethnicity, n (%)	
White or Caucasian	23 (79.3)
Asian	4 (13.8)
Hispanic	2 (6.9)
Black or African American	0 (0)
Relationship status, n (%)	
Currently married	14 (48.3)
Divorced	1 (3.4)
Single	13 (44.8)
Widowed	1 (3.4)
Level of education, n (%)	
High school diploma	2 (6.9)
Some college, no degree	5 (17.2)
Any college, graduate, or professional degree	22 (75.9)
Primary indication for surgery, n (%)	
Cancer	19 (65.5)
Primary types of abdominal procedures, n (%)	
Laparoscopic low anterior resection	11 (37.9)
Robotic-assisted low anterior resection	3 (10.3)
Laparoscopic colectomy	5 (17.2)
Open colectomy	2 (6.8)
Open colostomy revision or takedown	3 (10.3)
Ileostomy takedown	3 (10.3)
Open abdominal perineal resection	1 (3.4)
Robotic-assisted rectopexy	1 (3.4)
Prior pain and virtual reality use, n (%)	
Pain in the past week prior to surgery	16 (55.2)
Current opioid use for pain prior to surgery	3 (10.3)
Prior virtual reality use	2 (6.9)
Baseline scores, mean (SD)	
Pain Catastrophizing Scale (PCS)^a^	14.56 (14.21)
Health State Questionnaire – EQ-5D-5L^b^	71.10 (22.3)
Generalized Anxiety Disorder 7-item (GAD-7)^c^	5.56 (5.9)
Patient Health Questionnaire-8 (PHQ-8)^d^	5.03 (5.5)

a Pain Catastrophizing Scale consists of 13 items, with each item on a scale from 0 to 4 based on their thoughts when experiencing pain. The total score can range from 0 to 52, with higher scores indicating greater levels of pain catastrophizing.

b The EuroQol 5-Dimension 5-Level questionnaire uses a Visual Analog Scale (VAS), where the end points are labeled as the “best imaginable health state” and the “worst imaginable health state.” The VAS score ranges from 0 to 100, with the higher score indicating higher perceived health.

c Generalized anxiety disorder 7-item scale is a self-reported questionnaire used to assess the severity of anxiety symptoms with each item scored from 0 (not at all) to 3 (nearly every day). The total score ranges from 0 to 21, with higher scores indicating higher anxiety levels.

d The Patient Health Questionnaire-8 measures the severity of depressive symptoms. Each item is scored on a scale from 0 to 3. The sum of all items is a range of 0 to 24, with a higher score indicating greater levels of depressive symptoms.

### Acceptability

The mean duration of IVR use in the initial session for 29 participants was 19.14 (SD 7.67) minutes. Moreover, 19 participants additionally completed a second session, during which the mean usage time was 16.78 (SD 6.13) minutes (Table 2). The most common IVR experiences chosen by study participants were guided travel, followed by mindfulness and meditation, with nearly half of the participants choosing more than 1 IVR experience during a single session. The results indicated high perceived usability of IVR in this sample as demonstrated by a high mean SUS score of 88.10 (SD 6.15). SUS scores above 68 are considered above average and are an indicator of good usability [41]. Individual adjusted raw mean scores for each SUS item were generally >3, indicating positive usability for each SUS item statement (adjusted items ranged from 0 to 4, with 4 as more desirable per each item). Higher scores after adjustment indicate better usability (Table 3).

**Table 2.** Mean time spent using immersive virtual reality and content selection.

Usage time and content selection	1 day after surgery (N=29)	2 days after surgery (n=19)
Time spent in IVR^a^ (min), mean (SD; range)	19.14 (7.67; 6-30)	16.78 (6.13; 3-30)
Participant IVR content selection^b^, n (%)		
Guided travel	20 (68.9)	10 (52.6)
Mindfulness and meditation	7 (24.1)	5 (26.3)
Arctic cold and/or underwater	4 (13.7)	3 (15.8)
Forests and/or wildlife	2 (6.8)	1 (5.3)
Games	1 (3.4)	1 (5.3)

a IVR: immersive virtual reality.

b Participants had the option to choose as many experiences as desired, up to a maximum of 30 minutes of use per session, in any of the content categories offered within the preloaded software library.

**Table 3.** System Usability Scale (SUS) ratings.

SUS^a^ adjusted raw score per item (N=29)	Score, mean (SD)	Range
I think that I would like to use this system frequently.	3.5 (0.58)	2‐4
I found the system unnecessarily complex.	3.8 (0.77)	0‐4
I thought the system was easy to use.	3.7 (0.47)	3‐4
I think that I would need the support of a technical person to be able to use this system.	3.2 (0.85)	1‐4
I found the various functions in this system were well integrated.	2.48 (0.87)	1‐4
I thought there was too much inconsistency in this system.	3.7 (0.66)	2‐4
I would imagine that most people would learn to use this system very quickly.	3.6 (0.50)	3‐4
I found the system very cumbersome to use.	3.9 (0.37)	2‐4
I felt very confident using the system.	3.7 (0.47)	3‐4
I needed to learn a lot of things before I could get going with this system.	3.8 (0.41)	3‐4

a SUS: System Usability Scale.

The original responses are given on a Likert scale from 1 (strongly disagree) to 5 (strongly agree) for each of the 10 items. After adjusting the scale of negatively worded questions (items 2, 4, 6, 8, and 10) by subtracting their scores from 5 and for positively worded questions (items 1, 3, 5, 7, and 9) obtained by subtracting 1, adjusted scores will range from 0 to 4. After adjustment, “0” represents a negative usability experience as related to the item statement and “4” represents a positive usability experience for each item. Higher scores after adjustment indicate better usability (adapted from the study by Brooke [42]).

Study participants reported an overall positive experience with using IVR as indicated in the survey questionnaire (Table 4). All study participants marked responses of “agree-4” or “strongly agree-5” to the statement “I liked the virtual reality experience.” Most of the study participants (27/29, 93.1%) also marked “agree-4” or “strongly agree-5” that IVR improved their postoperative pain. Furthermore, most participants agreed that they would use IVR again for pain (26/29, 89.6%) or anxiety (25/29, 86.2%). Finally, all participants (N=29) marked “strongly agree” to the statement, “I would recommend virtual reality to other older surgical patients.”

**Table 4.** User experience survey responses (N=29).^a^

User experience item	Scores, mean (SD)
Would recommend VR^b^ to others	5.0 (0.0)
Would use VR again for pain	4.8 (0.4)
I liked the VR experience	4.8 (0.7)
Would use VR again for anxiety	4.7 (0.7)
The audio sound was pleasant	4.6 (0.6)
VR improved my pain	4.5 (0.8)
The image quality was pleasant	3.8 (0.8)
The headset was comfortable	3.1 (1.4)

a Participants rated each statement on a 5-point Likert scale (1=totally disagree, 2=disagree, 3=neutral, 4=agree, 5=totally agree). Mean scores and SDs are reported for each item and are ordered from highest to lowest mean rating.

b VR: virtual reality.

Nearly all participants (28/29, 96.6%) completed one or more IVR sessions without self-reported side effects (eg, dizziness, headache, eye strain, and nausea). One participant reported mild face and chest skin redness that occurred nearly 24 hours after the first IVR session. Upon further investigation, it was unclear as to the exact cause of the skin irritation, whether due to the IVR headset foam padding or related to recent medications as part of surgical care. Although unlikely related to the use of IVR equipment, the possible adverse event was reported out of an abundance of caution.

### Feasibility

To evaluate overall feasibility, we measured the rate of accrual and reasons for nonparticipation, as well as successful completion of IVR on the first and second day after inpatient abdominal surgery (Figure 1). Of the 55 potential participants assessed for eligibility, 7 individuals did not meet inclusion requirements during telephone screening, 7 declined to participate after learning more about the study, stating extreme anxiety over surgery (n=5) or fear of new technology (n=2) as the main reasons for nonparticipation, and 1 individual refused to state a reason for declining participation (Figure 1). The remaining participants (n=40) were deemed eligible and agreed to participate. Of the 40 participants, a total of 11 either dropped out or were excluded before the intervention was administered due to the following reasons: did not return the consent forms (n=2), decided to drop out of the study prior to the date of surgery for unspecified reasons (n=3), surgery was canceled (n=3), Intensive Care Unit admission directly from the operating room (n=1), and COVID-19–related reasons (n=2).

A total of 29 enrolled participants allocated to the intervention on the day following surgery all completed the first IVR session, with 19 additionally completing the second IVR session the next day. The most common reason for not completing a second IVR session the next day was typically due to a change in complexity of care or severe nausea or vomiting not related to IVR (n=5). All participants allocated to the first and second IVR sessions completed all baseline questionnaires, as well as all pre- and postintervention questionnaires, with no missing or unanswered items.

### Preliminary Clinical Impact of IVR on Pain and Relaxation

Significant improvements were observed in both pain and relaxation levels from pre- to post-IVR on both day 1 and day 2 following surgery (Table 5). The preliminary impact of IVR on pain levels was analyzed using a paired 2-sample *t* test. On day 1 following surgery, post-IVR mean pain levels showed a significant reduction as compared to pre-IVR pain levels, with a mean improvement of 2.65 (SD 2.0), representing approximately a 50% reduction in pain scores (95% CI 1.89-3.41; *P*<.001). Similarly, on day 2 following surgery, results indicated a significant decrease in pain levels from pre- to post-IVR, with a mean pain level decrease of 2.05 (SD 1.5; 95% CI 1.33-2.78; *P*<.001).

**Table 5.** Pre-to-post immersive virtual reality changes in self-reported pain and relaxation.

Pre-post IVR^a^ pain and relaxation levels	Mean (SD) or median (IQR)	95% CI	Test statistic	*P* value
Pain level day 1, N=29, mean (SD)			N/A^b^	.001
Pre-IVR	5.17 (2.1)	4.37‐5.97		
Post-IVR	2.51 (1.5)	1.91‐3.16		
Change	2.65 (2.0)	1.89‐3.41		
Pain level day 2, N=19, mean (SD)			N/A	.001
Pre-IVR	4.84 (1.6)	4.08‐5.60		
Post-IVR	2.79 (1.5)	2.06‐3.51		
Change	2.05 (1.5)	1.33‐2.78		
Relaxation level day 1, N=29, median (IQR)			Z=−4.6	<.01
Pre-IVR	3 (3-6)			
Post-IVR	8 (7-9)			
Change	—^c^			
Relaxation level day 2, n=19, median (IQR)			Z=−3.85	<.01
Pre-IVR	4 (4-5)			
Post-IVR	8 (7-9)			
Change	—			

a IVR: immersive virtual reality.

b N/A: not applicable.

c Not available.

Finally, participants reported a significantly higher level of relaxation immediately following IVR as compared to pre-IVR relaxation levels on both day 1 and day 2 following surgery. The Wilcoxon signed-rank test, applied due to the nonnormal distribution of relaxation scores, revealed significant findings. One day following surgery, the median relaxation score prior to using IVR was 3 (IQR 3-6), which increased to a median score of 8 (IQR 7-9) immediately following IVR usage, indicating a statistically significant improvement in relaxation levels from pre- to postintervention (Z=−4.6; *P*<.01). On the second day after surgery, the median score before intervention was 4 (IQR 4-5), which increased to a median relaxation score of 8 (IQR 7-9) following the intervention, demonstrating a significant improvement in relaxation scores from pre- to post-IVR use (Z=–3.85; *P*<.01).

## Discussion

### Principal Findings

Our study indicates that IVR is a feasible, acceptable, and well-tolerated intervention for postoperative pain management and relaxation in older adults in the initial days following elective major inpatient abdominal surgery. Participants reported high perceived usability and acceptance, with minimal side effects. IVR use resulted in statistically significant reductions in self-reported pain and improvements in relaxation from pre- to postintervention on both day 1 and day 2, with most participants indicating a willingness to use IVR again and recommend it to other older surgical patients. While prior studies have established the feasibility of IVR for preoperative anxiety and postoperative exercise in older adults undergoing colorectal surgery [49,50], our study is among the few to extend these findings to postoperative pain and relaxation across a wider range of elective abdominal procedures. These results support the potential of IVR as a nonpharmacologic option for pain management and relaxation in this population.

### Acceptability, Feasibility, and Tolerability

Our research both corroborates and diverges from existing literature on the application of IVR for managing pain and facilitating relaxation. Recent meta-analyses have suggested that IVR is efficacious in reducing acute pain following a variety of surgical and medical procedures [25,51]. However, except for total knee arthroplasty operations, there is a notable gap in research regarding the acceptability, feasibility, and tolerability of IVR among older surgical adults in the initial days following various types of major elective inpatient surgeries [52]. Some studies suggest that hospitalized older adults older than the age of 60 are more likely to decline participation in IVR studies, related to a lack of understanding or perceived usefulness of IVR [53,54]. Other research indicates a possibly lower acceptance of IVR among older adults in acute care settings due to negative attitudes and anxiety toward using technology [52,55]. Furthermore, except for those who developed postoperative clinical complications precluding the use of IVR, the dropout rate among our study participants was notably low at 12.5% (n=5). In both community and residential contexts, as well as within the scope of colorectal cancer care, existing research has indicated that the use of IVR as a positive distraction is both feasible and highly accepted among older adults for alleviating chronic pain in nonhospital settings [31,56-58]. Moreover, studies on the adoption of emerging technologies such as IVR suggest that older adults are generally more receptive to using new technology when they are offered a broad range of choices and the autonomy to select content and information according to their unique preferences [59-63].

Regarding the tolerability of IVR use in older adults, there have been minor reports of motion sickness and occasional discomfort with the IVR headset noted in prior research [52,64,65]. In our study, there were no reports of motion sickness during or after use. This finding may have been due to limiting each IVR session to a maximum of 30 minutes. A recent review of IVR use in older adults across a wide spectrum of nonsurgical settings found cybersickness (eg, dizziness, nausea, eye strain, etc) to be minimal across 39 studies [66,67]. Also related to the tolerability of IVR, in our study, 1 participant reported skin irritation, an adverse effect for which there is little to no existing research quantifying its incidence rate [68]. Finally, our user experience survey results suggest that while the overall satisfaction with the IVR system was positive, a significant number of participants expressed discomfort with the headset. This reported discomfort is consistent with prior research indicating that while older adults in community settings have found the headsets to be bulky and uncomfortable, their overall experience with IVR was enjoyable [30].

### Clinical Implications

The integration of IVR into the care of older adults following major abdominal surgery presents a promising avenue for enhancing pain management and relaxation strategies, with the potential to improve patient-reported outcomes, patient satisfaction, and the overall recovery experience. First, despite existing concerns about older adults’ willingness to engage with new technologies, our study found a high level of acceptability and tolerability of IVR for pain and relaxation. This finding suggests that with proper introduction and support, as we provided in our study, older adults might be open to using innovative technology such as IVR in the acute care setting as part of their care [28,69]. Second, our results hold particular significance in the surgical care of older adults, where IVR could act as a cost-effective and safe alternative or adjunct to conventional pain management approaches [9,70,71]. Some studies indicate that IVR retains the possibility to lessen opioid medication usage and its associated side effects [72,73]. In the last decade, government agencies and clinical professional organizations, including the American College of Surgeons’ Geriatric Surgery Verification program, have increased emphasis on the importance of incorporating opioid-sparing methods into pain treatment strategies, including nonpharmacological interventions, especially in vulnerable groups such as older adults [16,74]. The prominence of this push can also be exemplified through a recent ruling by the Centers for Medicare & Medicaid Services, indicating that one type of an integrated software or hardware IVR device may be eligible for Medicare insurance coverage [75]. In the near future, it is expected that improved insurance coverage will greatly increase the accessibility of such technologies for older adults as part of their pain management.

Furthermore, introducing IVR into the care of older adults may initially be met with uncertainty, by both clinicians and older adults, due to prevailing beliefs about technological proficiency or appropriateness in this age group. This view may originate from widespread societal perceptions on aging and technology, which commonly depict older adults as less skilled or less inclined to engage with new technology [76,77]. Such perspectives can unintentionally result in exclusion from IVR clinical trials or use in the clinical setting [78]. Thus, we may underestimate the older adults’ capacity to accept or learn new technology such as IVR. Personalized education, tailored training, and sufficient support are essential actions to enhance the adoption of IVR among older adults [60].

Finally, aligning IVR content with older adults’ personal interests, such as cultural and spiritual practices, as well as hobbies and previous life experiences, is vital for its wider acceptance in this demographic [30]. Future research is needed to investigate whether the level of pain or relaxation correlates with specific types of IVR content or exhibits a dose-response relationship based on duration and frequency of use. Finally, the insights gained from this study could pave the way for more extensive randomized controlled trials aimed at assessing the acceptance and overall efficacy of IVR in older adults undergoing a variety of major elective surgeries.

### Limitations

The findings of this study should be considered in view of certain limitations. The study design and limited sample size restrict the generalizability of the findings; therefore, final conclusions about the efficacy of IVR to improve pain and relaxation cannot be made. Additionally, this study was not designed or powered to reliably assess the impact of predictors such as age, gender, specific surgeries, or other baseline characteristics on study outcomes. Postoperative pain and relaxation outcomes are influenced by multiple perioperative factors that were not controlled in this initial feasibility study, including baseline pain levels prior to surgery, preoperative opioid consumption, extent of colorectal surgery, use of regional anesthesia or local blocks, and concurrent use of multimodal opioid-sparing medications. Moreover, the reliance on self-report introduces potential for bias and subjectivity in our findings. Although targeted recruitment efforts were undertaken to increase diversity, the study sample reflected the demographic composition of elective colorectal surgery patients treated within a single academic health care system during the study period. As a result, these findings may not fully capture the experiences of individuals from racially or ethnically diverse backgrounds, non-English speakers, those with lower educational attainment, or patients with frailty or cognitive impairment. This limitation highlights the importance of future multisite studies to more robustly evaluate the feasibility, accessibility, and broader applicability of IVR interventions in colorectal surgical populations. Also, because pain and relaxation outcomes were assessed only immediately before and after each IVR session, we were unable to determine the duration of the intervention’s effects beyond the short-term period. Given the short average postoperative length of stay in this cohort (just over 3 days), the intervention and assessments were necessarily limited to the immediate inpatient recovery window. It is key to note that although questionnaires were brief, repeated pre-post assessments across multiple IVR sessions may have contributed to respondent burden and fatigue in the immediate postoperative setting, although not mentioned by participants. Survey methodology literature suggests that repeated measures can affect response quality. Importantly, the assessment schedule remained feasible in this pilot study; however, larger and more longitudinal studies should explicitly evaluate participant burden related to repeated questionnaires and assessment frequency [79]. Finally, the IVR intervention was not standardized, allowing variation in both content selection and duration of use. Given that some participants selected briefer immersive content experiences while others completed longer sessions, it remains unclear whether shorter exposure reflected content preference, postoperative fatigue, discomfort, or other barriers to sustained use. Future studies should consider controlling content type, duration, and frequency of use to better assess the impact of IVR on acceptability, feasibility, and clinical outcomes.

### Conclusion

This study supports the feasibility, acceptability, and tolerability of IVR as a potential intervention for postoperative pain among older adults following elective inpatient abdominal surgery. Our findings add to the growing body of evidence supporting nonpharmacological approaches for postoperative pain management. While our preliminary findings are promising, larger-scale studies are needed to confirm the acceptance and efficacy of IVR as a postoperative pain management intervention across more diverse populations of older adults, including those from underrepresented minority groups and individuals with physical or cognitive limitations. Importantly, this study highlights the potential of IVR to enhance patient-reported outcomes and improve the perioperative care experience for a demographic that is often considered vulnerable and is frequently underrepresented in technology-based research.

## Abbreviations

CONSORT: Consolidated Standards of Reporting Trials
GAD-7: Generalized Anxiety Disorder 7-item scale
HMD: head-mounted display
IVR: immersive virtual reality
NRS: Numeric Rating Scale
PCS: Pain Catastrophizing Scale
PHQ-8: Patient Health Questionnaire-8
SPMSQ: Short Portable Mental Status Questionnaire
SSQ: Simulator Sickness Questionnaire
SUS: System Usability Scale
VAS: Visual Analog Scale
VR-CORE: Virtual Reality Clinical Outcomes Research Experts
